# Split-bolus versus triphasic multidetector-row computed tomography technique in the diagnosis of hepatic focal nodular hyperplasia: a case report

**DOI:** 10.1186/1752-1947-8-425

**Published:** 2014-12-14

**Authors:** Michele Scialpi, Luisa Pierotti, Sabrina Gravante, Irene Piscioli, Teresa Pusiol, Raffaele Schiavone, Alfredo D’Andrea

**Affiliations:** 2 Department of Surgical and Biomedical Sciences, Division of Radiology 2, Perugia University, S. Maria della Misericordia Hospital, S. Andrea delle Fratte, 06134 Perugia, Italy; 3 Department of Radiology, Budrio Hospital, Azienda USL Bologna, 40054 Budrio, Italy; 4 Department of Pathology, S. Maria del Carmine Rovereto Hospital, Rovereto, Italy; 5 Division of Radiology, Meyer Pediatric Hospital, Firenze, Italy; 6 Division of Radiology, San Giuseppe Moscati Hospital, Aversa, Caserta, Italy

**Keywords:** Focal nodular hyperplasia (FNH), Liver, Multidetector-row CT (MDCT), Split-bolus technique, Triphasic technique

## Abstract

**Introduction:**

Computed tomography and magnetic resonance imaging are able to demonstrate and to diagnose hepatic focal nodular hyperplasia when a typical pattern of a well-circumscribed lesion with a central scar is present.

Our aim is to propose the split-bolus multidetector-row computed tomography technique as an alternative to the conventional triphasic technique in the detection and characterization of focal nodular hyperplasia to reduce the radiation dose to the patient.

To the best of our knowledge, this is the first report regarding the application of the split-bolus computed tomography technique in the evaluation of hepatic focal nodular hyperplasia.

**Case presentation:**

We describe a case of focal nodular hyperplasia of the liver in a 53-year-old Caucasian woman (weight 75Kg) with a colorectal adenocarcinoma histologically confirmed. An innovative split-bolus multidetector-row computed tomography technique was used that, by splitting intravenous contrast material in two boli, combined two phases (hepatic arterial phase and portal venous phase) in a single pass; a delayed (5 minutes) phase was obtained to compare the findings with that of triphasic multidetector-row computed tomography.

**Conclusions:**

Split-bolus multidetector-row computed tomography was able to show the same appearance of the lesion as the triphasic multidetector-row computed tomography technique.

This is the first case demonstrating the effectiveness of the split-bolus multidetector-row computed tomography technique in the detection and characterization of focal nodular hyperplasia with a significant reduction in radiation dose to the patient with respect to triphasic multidetector-row computed tomography technique.

## Introduction

Focal nodular hyperplasia (FNH) is the second most common benign tumor of the liver after hemangioma; its etiology is still unclear [1]. In most patients, the clinical course is silent and FNH is often discovered incidentally during radiologic imaging performed for other reasons. The lesion typically is well circumscribed with a central scar. Although FNH usually has no clinical significance, recognition of the radiologic characteristics of hepatic FNH is important to avoid unnecessary surgery, biopsy, and follow-up imaging [2, 3].

Triphasic helical computed tomography (CT) scans – hepatic arterial phase (HAP), portal venous phase (PVP) and delayed phase (DP) – represent an accurate technique in the characterization of typical FNH [4].

Split-bolus protocol is an innovative technique that, by splitting intravenous contrast medium into two boli and combining phase images in a single scan, is used in several CT applications [5, 6].

To the best of our knowledge the application of the split-bolus CT technique in the characterization of FNH of the liver has not been reported.

The aim is to demonstrate the effectiveness of the split-bolus multidetector-row CT (MDCT) technique in the characterization of FNH of the liver.

## Case presentation

A 53-year-old Caucasian woman with a colorectal adenocarcinoma, histologically confirmed, underwent preoperative triphasic MDCT of her chest-abdomen-pelvis, magnetic resonance imaging (MRI) and follow-up split-bolus MDCT. Triphasic and split-bolus MDCT were performed by Philips Brilliance 64-detector row scanner (Philips Healthcare, Best, The Netherlands).

The triphasic MDCT protocol consisted of unenhanced images and a HAP (started 40 seconds after the injection of the contrast medium or 20 seconds after threshold of 150 Hounsfield units, HU, in her thoracic-abdominal aorta by using a bolus-tracking technique) of her upper abdomen, a PVP (started 40 seconds after the end of HAP acquisition) of her chest-abdomen-pelvis and a DP (after 5 minutes to the start of injection) of her upper abdomen. A bolus administration of intravenous contrast material (iopamidole, Iopamiro^®^; Bracco, Milan, Italy; 1.5mL per kg body weight, containing 370mg iodine/mL) was injected at 4.0mL/second via an injector at a rate of 4.0mL/second from an antecubital vein using an 18 gauge needle.

**Figure 1:**
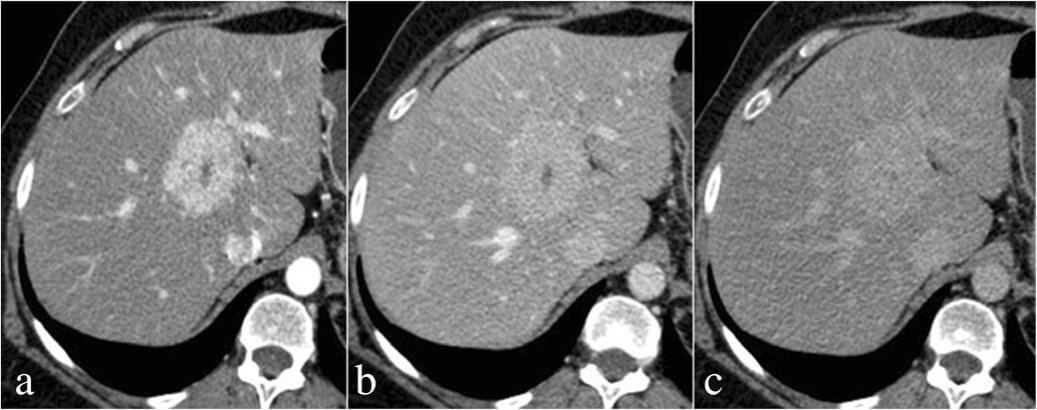
**Triphasic 64-detector row computed tomography findings of focal nodular hyperplasia in a 53-year-old woman.** Arterial phase contrast-enhanced computed tomography scan shows intense homogeneous enhancement with hypodense focal central scar **(a)**; contrast-enhanced computed tomography scan during the portal venous phase shows lesion exhibiting rapid contrast material washout being slightly hypo-attenuated compared with surrounding liver **(b)**; delayed phase contrast-enhanced computed tomography scan shows lesion as isodense and persistent enhancement of central scar **(c)**.

Triphasic MDCT demonstrated a lesion (5.5cm in maximum diameter) in the right lobe of her liver with diffuse immediate homogeneous hyperdense enhancement on HAP, washout of contrast medium becoming isodense to her liver on PVP and DP at 5 minutes. Scar was hypodense to the liver on unenhanced phase, showing a progressive enhancement throughout the three phases with maximum peak on DP (Figure 1). A diagnosis of FNH was suggested.

Her radiation dose – the effective dose (sievert, Sv) was calculated using the following equation: effective dose = k × dose-length-product; k = 0.015 (conversion coefficient) [7] – obtained during triphasic MDCT was 38.87mSv. The total number of the images was 878.

MRI with liver-specific hepatobiliary contrast agent gadoxetic was done, showing a hypervascular soft tissue lesion consistent with a diagnosis of FNH. Laboratory data were all within the normal range. Liver and other specific tumor markers (alpha-fetoprotein, CA 19–9, carcinoembryonic antigen) turned out negative. At follow-up after 12 months from the initial triphasic MDCT, a split-bolus MDCT protocol was used.

**Figure 2:**
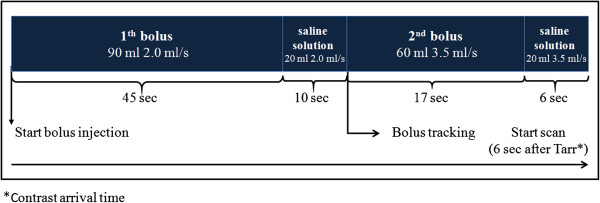
**Schematic view of split-bolus 64-detector row computed tomography scanning of the chest and abdomen of a 53-year-old Caucasian woman (weight 75kg) with a colorectal adenocarcinoma and incidental liver focal nodular hyperplasia.** First bolus at the start of bolus injection, or time zero: 90mL (1.2mL/kg) of contrast medium at 2.0mL/second, followed by 20mL of saline solution at the same flow rate, is injected to obtain adequate hepatic enhancement during the portal venous phase. Second bolus: 60mL of contrast medium at 3.5mL/second followed by 20mL of saline solution at the same flow rate to obtain hepatic arterial phase. A single contrast-enhanced acquisition from the pulmonary apex to the pubic symphysis was acquired. A circular region of interest of the bolus-tracking technique was placed in the descending aorta. At the start of the second bolus contrast medium injection, the scan started cranio-caudally after a delay of 6 seconds from the arrival of the contrast medium into the aorta. Abbreviations: sec, second(s); Tarr, arrival time of the contrast medium into the aorta.

The split-bolus MDCT protocol consisted of unenhanced low-dose of her upper abdomen; a single acquisition of the chest-abdomen-pelvis after intravenous injection of 150mL of contrast medium (370mgI/mL, iopamidole, Iopamiro^®^; Bracco, Milan, Italy), split by an automatic power injector (Stellant, CT; Medrad, Indianola, Pa, USA) into two boluses (Figure 2).

Using the scout film, a scan range from her pulmonary apex to her pubic symphysis was determined. Then a circular region of interest of the bolus-tracking technique (raising the threshold value at 500 HU) was placed in the descending aorta. At the start of the second bolus contrast medium injection, the scan started cranio-caudally after a delay of 6 seconds from the arrival of the contrast medium into her aorta. The inherent 6-second delay in the bolus-tracking technique is necessary to move the scan table to the start of the scan, give breath-hold instructions to the patient, and tune the gantry parameters.

A single contrast-enhanced acquisition of the chest-abdomen-pelvis was acquired, resulting in a simultaneous contrast enhancement of her arterial and venous system.

**Figure 3:**
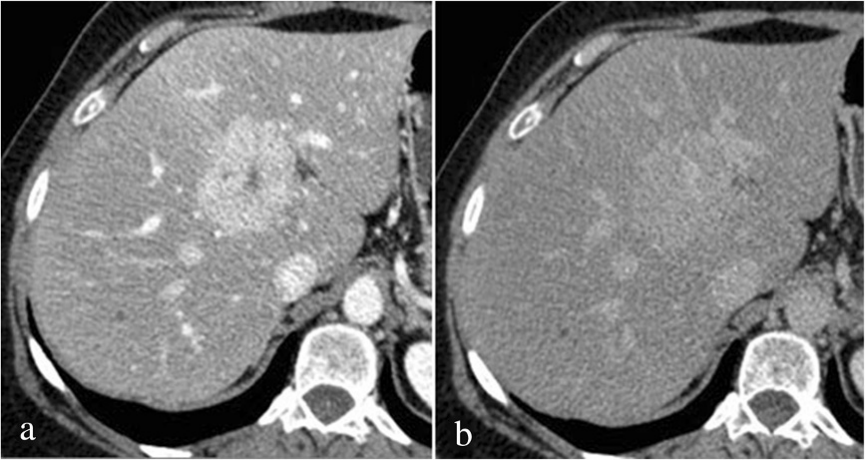
**Single-pass 64-detector row computed tomography findings of focal nodular hyperplasia in 53-year-old woman.** Mixed phase (hepatic arterial phase/portal venous phase during hepatic enhancement) shows intense homogeneous enhancement with hypodense focal central scar **(a)**; on delayed phase **(b)** the lesion appears substantially isodense to liver parenchyma with persistent enhancement of central scar.

Split-bolus MDCT showed the typical hyperdense enhancement of the lesion with hypodense central scar at the combined phase images (HAP/PVP during hepatic enhancement) and isodensity with hyperdensity of central scar at DP (5 minutes; Figure 3).

The dose of radiation obtained during split-bolus CT was 22.78mSv. The total number of the images was 637.

**Figure 4:**
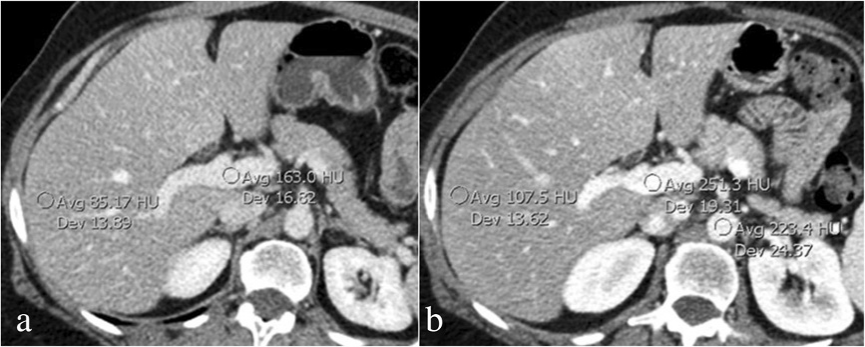
**Circular region of interest measurements of portal vein and liver parenchyma performed during portal venous phase (a) of the triphasic computed tomography and during combined phase (b) of the single-pass split-bolus computed tomography.** Mixed phase (hepatic arterial phase/portal venous phase during hepatic enhancement) of the split-bolus multidetector-row computed tomography shows a higher attenuation of the portal vein and liver parenchyma with respect to that of the portal venous phase of the triphasic multidetector-row computed tomography protocol. Abbreviations: Avg, average; Dev, standard deviation; HU, Hounsfield unit.

Split-bolus MDCT demonstrated no differences in quantitative analysis in HU on liver parenchyma, portal vein and aorta with respect to triphasic MDCT technique (Figure 4).

For triphasic and split-bolus MDCT protocol, the following acquisition parameters were used: slice thickness 2.5mm; gantry rotation speed 0.75 seconds; reconstruction index 1.25; pitch 0.935:1; 120 peak kilovoltage and automatic tube current (mA) was set on the basis of the patient’s weight using z-axis modulation. MDCT examinations were completed with sagittal, coronal and curved multiplanar reconstructions.

Images were transferred to an external workstation (Advantage Workstation 4.2, GE Healthcare, Milwaukee, USA and Magic View, Philips Medical Systems, Best, The Netherlands) and stored in a picture archiving and communication system (PACS, Agfa Healthcare, Impax).

## Discussion

Focal nodular hyperplasia (FNH) of the liver remains a largely asymptomatic disease that patients often only discover after vague abdominal symptoms or from imaging for another medical concern [8].

Accurate diagnosis is important in FNH as it dictates the course of treatment. In most cases, FNH usually presents with classic CT and MRI features, helping to narrow the differential diagnosis and negating the need for biopsy or further studies [9–11].

In general, FNH of the liver appears as a solitary nodule smaller than 5cm in diameter, usually lobulated and well circumscribed, although unencapsulated [12, 13]. On cut section the pathognomonic feature is the presence of a white depressed area of fibrosis often seen in the center with broad strands radiating from it to the periphery in a stellate configuration. The central scar contains thick-walled vessels that provide excellent arterial blood supply to the lesion and, therefore, these tumors are usually homogeneous (with internal necrosis and hemorrhage being extremely rare) [12, 13]. On microscopic examination, all the components of the normal liver lobule are present. The cellular morphology and relationship between hepatocytes and bile ducts are essentially those of normal liver, both by light and by electron microscopic criteria.

Triphasic CT has gained acceptance as the preferred technique for the evaluation of a wide range of liver lesions, like FNH, as demonstrated in the literature [3, 8]. Most FNHs are isodense or slightly hypodense to the liver on unenhanced CT [13]; when isodense to the liver, the lesions may be detectable only because of mass effect [3].

Typical FNH, during the HAP shows an immediate and intense enhancement (96%) relating to the hypervascularity of the tumor, with rapid washout of contrast medium becoming isodense to the liver during the peak of PVP and delayed images [14]. Scars is hypodense to the liver on unenhanced and early contrast-enhanced images, showing a progressive enhancement throughout the HAP and PVP with maximum peak in DP caused by the presence of abundant myxomatous stroma [14].

An enhancing vessel may be seen in the scar on arterial phase imaging, representing the intratumoral portion of the feeding artery. On occasion, contrast-enhanced images reveal hypoenhancing radiating fibrous septa. These septa usually divide the tumor into sections because they radiate from the central scar toward the periphery.

On triphasic helical CT examination hepatomas, hypervascular metastases, FNH and adenomas may all appear similar. Gadoxetate disodium-enhanced MRI when the standard series are combined with the hepatobiliary phase shows high accuracy for diagnosis of FNH and to differentiate it from adenoma in lesions larger than 2cm [15, 16].

To the best of our knowledge there are no reports concerning the use of the split-bolus MDCT technique in the detection and characterization of FNH.

*Spli-bolus technique* is an innovative CT imaging investigation that combines the HAP and the PVP hepatic enhancement in a single acquisition allowing the identification of hypodense, hyperdense and mixed lesions. Similar to triphasic MDCT, a DP of the upper abdomen further helps in the characterization of the lesion [17].

Split-bolus technique consists in splitting intravenous contrast medium into two boli, performing MDCT acquisition in a single pass. The rationale is similar to that of a CT urography protocol [18], with the same goal to standardize a split-bolus MDCT protocol to ensure diagnostic efficacy and reduce radiation dose [19, 20].

To understand the split-bolus MDCT protocol and MDCT appearance of the reported FNH, it is essential to know the peak enhancement time of main portal vein and liver related to patient weight, determined by the first bolus of contrast medium.

Erturk *et al.* [21] reported relationships between patient weight, peak enhancement time of main portal vein and liver. In patients with weight ranging from 60 to 70kg, at 40 and 45 seconds of contrast medium injection duration time, an optimal enhancement peak of the main portal vein (212.4 ±20.4 HU and 206.5 ±19.2 HU) and of the liver (59.8 ±9.6 HU and 58.9 ±7.8 HU) respectively was obtained.

In our split-bolus MDCT protocol, using for first bolus 90mL of contrast medium (1.2mL/kg of 370mgI/mL) with a fixed flow rate of 2mL/second and duration injection time of 45 seconds in a patient weighing 75kg, an optimal attenuation in HU of the aorta, the liver parenchyma and the main portal vein was obtained with results similar to that of the standard triphasic MDCT protocol. In addition, optimal patients’ body-weight-tailored dose of first bolus of contrast medium for the liver has been determined based on a definition of the dose which can give adequate hepatic enhancement (more than 50 HU increase from unenhanced baseline HU of the liver) during hepatic parenchymal phase [22, 23]. The second bolus of 60mL fixed of contrast medium at a 3.5mL/second flow rate ensured an adequate HAP demonstrating the hypervascularity of the FNH and hypodensity of the central scar and its maximum peak of enhancement on DP.

In our experience, the split-bolus MDCT protocol resulted in a reduction in the radiation dose with respect to the standard triphasic MDCT protocol (respectively 22.78mSv versus 38.87mSv; reduction in dose of approximately 40%), without compromising image quality. In addition the new protocol reduces the number of data sets (637 images for split-bolus versus 878 images for triphasic technique; reduction in the images of 27.5%). Instead of obtaining two scans separately at the HAP and PVP during hepatic enhancement, we obtained a single combined-phase scan.

The unenhanced and DP phases were performed with both the triphasic and split-bolus MDCT technique for an adequate comparison between the two techniques and to demonstrate the effectiveness of split-bolus MDCT in characterization of the lesion.

## Conclusions

In conclusion, this original case demonstrates the high accuracy of split-bolus MDCT technique in the diagnosis of FNH in a patient with a tumor. The split-bolus MDCT technique confirmed the typical features of FNH as did the triphasic MDCT technique but with a reduction in radiation dose (approximately 40%) to the patient and data stored.

The split-bolus MDCT technique can be performed in the follow-up of patients with known hepatic benign neoplasm and more research with large series is necessary in order to confirm it.

Split-bolus MDCT was able to show the same appearance of the lesion as the triphasic MDCT technique.

## Consent

Written informed consent was obtained from the patient for publication of this case report and any accompanying images. A copy of the written consent is available for review by the Editor-in-Chief of this journal.

## Abbreviations

CT: Computed tomography
DP: Delayed phase
FNH: Focal nodular hyperplasia
HAP: Hepatic arterial phase
HU: Hounsfield unit
MDCT: Multidetector-row computed tomography
MRI: Magnetic resonance imaging
PVP: Portal venous phase
Sv: sievert.
